# Microwave-synthesized freestanding iron-carbon nanotubes on polyester composites of woven Kevlar fibre and silver nanoparticle-decorated graphene

**DOI:** 10.1038/srep40386

**Published:** 2017-01-11

**Authors:** Ankita Hazarika, Biplab K. Deka, DoYoung Kim, Kyungil Kong, Young-Bin Park, Hyung Wook Park

**Affiliations:** 1 Department of Mechanical engineering, Ulsan National Institute of Science and Technology, Ulsan 44919, Korea

## Abstract

We synthesized Ag nanoparticle-decorated multilayered graphene nanosheets (Ag-graphene) from graphite nanoplatelets and silver nitrate through 90–100 s of microwave exposure, without the use of any mineral acids or harsh reducing agents. Fe nanoparticle-decorated carbon nanotubes (Fe-CNTs) were grown on polypyrrole (PPy) deposited on woven Kevlar fibre (WKF), using ferrocene as a catalyst, under microwave irradiation. Fe-CNTs grown on WKF and Ag-graphene dispersed in polyester resin (PES) were combined to fabricate Ag-graphene/Fe-CNT/PPy-coated WKF/PES composites by vacuum-assisted resin transfer moulding. The combined effect of Fe-CNTs and Ag-graphene in the resulting composites resulted in a remarkable enhancement of tensile properties (a 192.56% increase in strength and 100.64% increase in modulus) as well as impact resistance (a 116.33% increase). The electrical conductivity significantly increased for Ag-graphene/Fe-CNT/PPy-coated WKF/PES composites. The effectiveness of electromagnetic interference shielding, which relies strongly on the Ag-graphene content in the composites, was 25 times higher in Ag-graphene/Fe-CNT/PPy-coated WKF/PES than in neat WKF/PES composites. The current work offers a novel route for fabricating highly promising, cost effective WKF/PES composites through microwave-assisted synthesis of Fe-CNTs and Ag-graphene.

As most electronics now-a-days are wireless, electromagnetic interference (EMI) has been receiving increasing attention due to its impact on EMI-sensitive electronic devices and telecommunication. Shielding of these unnecessary electromagnetic (EM) waves has become a big concern for many applications, including those in military and commercial sectors^1^,^2^. The attenuation of EM waves can be achieved by reflection or absorption of received radiation^3^. Generally, EM waves are shielded by means of conventional metal barriers, which possess drawbacks of increased weight, reduced flexibility, susceptibility to corrosion, and restricted effectiveness of tuning the EM radiation^4^. Conductive polymer composites (CPCs) have gained attention due to their light weight, flexible design and corrosion-resistant properties^5^,^6^. Carbon-based nano-sized filler materials such as carbon nanofibers (CNFs), graphite, carbon black (CB), and carbon nanotubes (CNTs) have been widely used in polymer composites for EMI shielding purposes, mechanical reinforcement as they have superior corrosion resistance, are electrically conductive and have low weight densities^7^,^8^,^9^. CNTs, in particular are an excellent candidate to produce high-performance conductive composites that are required for EMI shielding, due to their high aspect ratio, surface area, excellent dielectric properties, high conductivity and superior mechanical properties^10^,^11^. It is potential nano reinforcement in fiber-reinforced polymer composite that effectively helps to transfer load on application of stress. The high aspect ratio of multiwall carbon nanotubes (MWCNTs), for example, lowers their percolation threshold compared to metal nanoparticles, which results in a high electrical conductivity^12^. However, the shielding mechanism by absorption is lacking, as magnetic dipoles are absent in MWCNTs. Therefore, the use of ferromagnetic compounds like Fe, Co, and Ni nanoparticles could be highly promising for EMI shielding. Physical mixing or decorating CNTs with such metal nanoparticles are two ways to form composites. However, due to Van der Waals forces between the nanotubes, a high CNT loading fraction leads to their agglomeration, which hampers their reinforcement effectiveness. The enhancement in the mechanical properties of the composites primarily depends on an efficient load transfer mechanism between the polymer matrix and the nanotubes and the load transfer between the nanotubes themselves. A homogenous dispersion of CNTs at high loading fractions may be achieved in polymer composites, if CNTs can be directly grown on the surface of the base fibres. Among the different synthetic methods for the growth of CNTs, such as chemical vapour deposition (CVD), templated growth, lithography, and arc discharge, microwave-assisted growth is both very fast and economical, while all other methods are tedious, need costly apparatus, require an inert gas or are multistep processes^13^,^14^.

Graphene, a unique and very promising material, is a one-atom thick 2-D aromatic sheet consisting of sp^2^-bonded carbon atoms. This exclusive structure leads to high mechanical stiffness and exceptional electrical conductivity and thermal properties that make it highly suitable for diverse potential applications including EMI shielding and polymer reinforcement^15^,^16^. Different methods have been reported for the synthesis of graphene sheets, such as thermal expansion of graphite^17^, chemical reduction of exfoliated graphite oxide (GO)^18^, and micromechanical cleavage and epitaxial growth on SiC surfaces^19^. However, the synthesis of graphene sheets through these methods is very time consuming and involves high temperatures. Furthermore, graphene production through chemical methods mostly requires strong mineral acids and oxidizers like H_2_SO_4_, KMnO_4_^20^, formic acid or carboxylic acid^21^, while overuse of organic solvents for successive exfoliation of graphene sheets is harmful to the environment. As a result, microwave-initiated techniques for the synthesis of graphene nano-sheets have been considered, since they are eco-friendly, cheap, and allow for short processing times. By incorporating suitable metal precursors, such techniques also allow the preparation of graphene nanosheets decorated with metal nanoparticles, which further enhances their mechanical stiffness and electrical conductivity, making them effective for EMI shielding applications.

Kevlar is notable for its high strength, tenacity, and impact resistance, and has major applications as a body and vehicle armour material, as well as a structural composite material in automobiles and aircrafts^22^. The growth of metal nanoparticle-decorated CNTs on the surface of its fibres not only gives Kevlar improved strength, but also enhances its electrical conductivity. Electrically conductive Kevlar composites may help protect electronic devices and aircrafts from electromagnetic pollution by acting as an EMI shield.

Hereby, the present study focuses on the combined effect of Fe-CNT and Ag-graphene on the tensile properties, impact response, electrical conductivity and EMI shielding effectiveness of the composites. Polypyrrole (PPy) was initially, deposited on the surface of a woven Kevlar fibre (WKF) sheet through *in-situ* polymerization of pyrrole. Fe nanoparticle-decorated CNTs (Fe-CNTs) were grown on the surface of the PPy-deposited WKF within a very short period of time (15–30 s) via a unique microwave-assisted method. The conducting layer of PPy on the surface of insulating WKF helps with the rapid increase of the temperature above 1100 °C to create arcs and sparks when exposed to microwave radiation. Ag nanoparticle-decorated multilayered graphene nanosheets (Ag-graphene) were also synthesized separately from graphite nanoplatelets (XGnP) by a similar eco-friendly microwave technique. Composites of polyester resin (PES) with Fe-CNTs grown WKF were prepared through a vacuum-assisted resin transfer moulding (VARTM) technique. Prior to the VARTM process, Ag nanoparticle-decorated graphene layers were dispersed in the resin by sonication. The schematic diagram for the preparation of the composites is shown in Fig. 1.

## Results and Discussion

**Figure 2:**
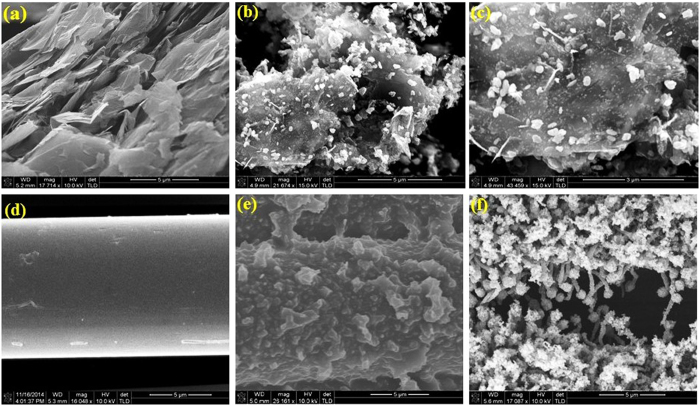
Scanning electron microscopy (SEM) micrographs of: (**a**) graphene nanoplatelets (XGnP), (**b,c**) Ag nanoparticle-decorated multilayered graphene at high and low resolution, (**d**) WKF, (**e**) PPy-coated WKF, and (**f**) Fe-CNTs grown on PPy-coated WKF.

The scanning electron microscopy (SEM) micrographs of stacked layers of XGnP, the precursor for the formation of graphene nanosheets, are shown in Fig. 2(a). After microwave irradiation, Ag nanoparticles grafted on the surface of graphene nanosheets could be observed (Fig. 2(b,c)). Under microwave irradiation, lightning and release of gases occurred, which caused exfoliation of the XGnP. The synthesis of multilayered graphene nanosheets involved mainly: 1) oxidation, 2) intercalation, and 3) expansion. The oxidation reaction proceeded in two steps: initially, ammonium persulphate decomposed under microwave irradiation to form oxide radicals that started the oxidation reaction at the ends of the graphite sheets; secondly, further exposure to microwave radiation resulted in oxidation in the inner layers of XGnP by hydrogen peroxide. Subsequently, urea and ammonia decomposed, releasing gaseous nitrogen, carbon monoxide and carbon dioxide that rapidly expanded the stacked layers along the c-axis to yield multilayered graphene nanosheets. Furthermore, urea sublimation yielded azine and cyanuric acid compounds, which acted as efficient reducing agents^23^. This eliminated the need for a final reduction step with environmentally harmful hydrazine or sodium borohydrate to form graphene from graphene oxide. Finally, due to the microwave irradiation, silver nitrate decomposed, aiding the nucleation of Ag nanoparticles on the graphene nanosheets’ surfaces.

Uncoated WKF had a uniform, smooth surface (Fig. 2(d)), whereas *in-situ* PPy-deposited WKF had a rough and uneven surface (Fig. 2(e)), with PPy granules visible on the surface of the WKF fibres. Upon exposure of PPy-coated WKF, blended with ferrocene (1:1), toluene and hexane, to microwave irradiation, Fe-CNTs grew abundantly on its surface. The CNTs, which contained Fe nanoparticles in their tips, were randomly oriented around the fibres (Fig. 2(e)). The PPy absorbed the microwave radiation, acted as a heating ground, and was converted to graphitic nanocarbons^24^. Free Fe atoms and cyclopentadienyl (C_5_H_5_) were formed because of the rapid rise in temperature. The formed Fe atoms might be in a molten state that may later pave the way for the formation of Fe nanoparticles through condensation of the molten Fe atoms. The sources of C required for the CNT growth were cyclopentadienyl, toluene and hexane. The C atoms remained dispersed across the Fe nanoparticles above the substrate. Graphitic walls, which later commenced the CNT growth, formed through arrangement of C atoms that precipitated from the Fe nanoparticles^25^.

**Figure 3:**
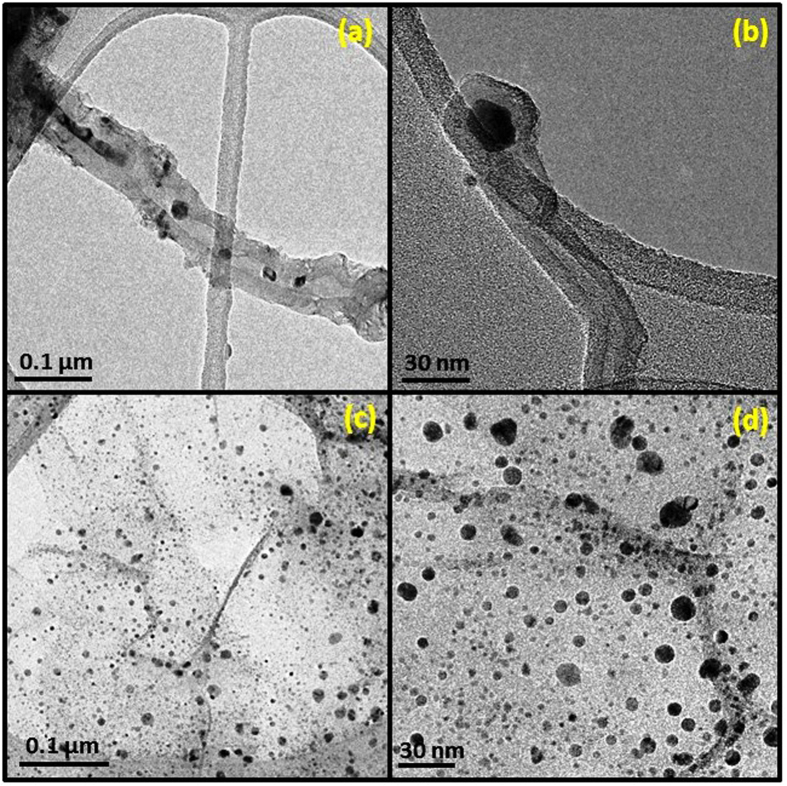
Transmission electron microscopy (TEM) micrographs of: (**a**,**b**) Fe-CNT, (**c,d**) Ag-graphene.

The transmission electron micrographs (TEM) of Fe-CNT and Ag-graphene are shown in Fig. 3. The TEM analysis revealed that the CNTs entrapped the Fe nanoparticles in between each nanotubes and also at its tip (Fig. 3(a,b)). The Fe nanoparticles that might have existed in the fused state at the beginning were consumed by the nanotubes due to the capillary effect promoted by their growth from the graphitic walls^26^. There are several parameters accountable for encapsulation of Fe nanoparticles inside the CNTs that comprises of: the viscosity of the fused Fe nanoparticles; the frictional force between the internal walls of a CNT and the entrapped Fe nanoparticles; capillary force of the nanotubes during their growth; the tension between different forces; and pressure on the Fe nanoparticles due to the growth of the nanotubes^25^. In some cases, the fused Fe atom develop into elongated structure within the nanotubes instead of forming smaller nanoparticles (Fig. 3a). The homogeneous distributions of Ag nanoparticles on the surface of graphene nanosheets were observed from the Fig. 3(c,d). The uniformly dispersed smooth waves of graphene sheets has been observed upon which the spherical morphological Ag nanoparticles were attached onto its surface and no nanoparticles were noticed segregated from the graphene nanosheets. The adhesion of Ag nanoparticles inhibited the aggregation of graphene sheets and increased the separation gap between each sheet of graphene layers. The diameters of most of the Ag nanoparticles were in the range of 5–20 nm. The graphene sheets controlled the morphologies of Ag nanoparticles to become spherical in shape and had a crucial role during nucleation and stabilization of the nanoparticles that assisted the growth of smaller sized nanoparticles^27^.

**Figure 4:**
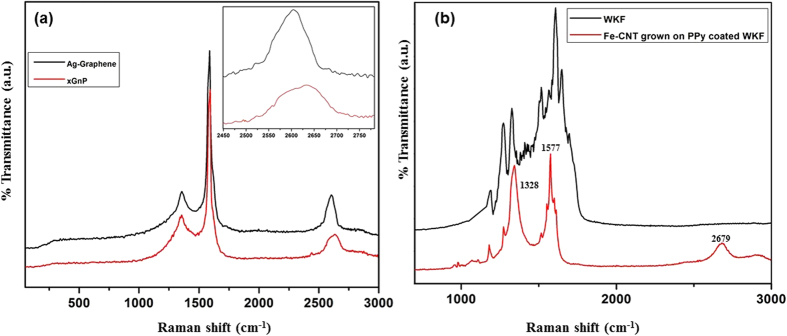
Raman spectra of: (**a**) XGnP and Ag nanoparticle-decorated graphene, (**b**) WKF and Fe-CNT grown on PPy-coated WKF.

Figure 4(a) shows the Raman spectra of XGnP and Ag-graphene, which reveal that both graphene and XGnP have similar D bands appearing at 1349 cm^−1^. This D band, a result of the one phonon lattice vibration process, is generally very weak in the spectra of both graphite and graphene. If it is intense, it indicates that the synthesized material contains many defects. Thus, the amount of defects present in graphene is directly proportional to the intensity of the D band^28^. Here, the amplitude of the D band was the same for both XGnP and Ag-graphene, indicating that the synthesized Ag-graphene was not defect laden. The noticeable dissimilarity between the spectra of graphite nanoplatelets and Ag-graphene was the position and intensity of the 2D band. The ratio of intensity between G and 2D band (I_G_/I_2D_) differentiates between less exfoliated graphite and few-layered graphene layers^29^. A slight shift to a lower wave number and small increase in intensity of the 2D band were observed in case of Ag-graphene i.e. the ratio I_G_/I_2D_ decreased for multilayered graphene indicating good exfoliation of graphite layers. The peak shift in graphene was a result of interactions between the stacked graphene layers, which tends to shift bands to lower wave numbers. The intensity of the 2D band was less pronounced compared to single layer graphene nanosheets but higher than that of XGnP. The 2D band was composed of two peaks in the XGnP spectra of XGnP (2D-1 and 2D-2), as shown in Fig. 4(a) (insets),whereas the 2D band of Ag-graphene showed a single peak due to the thinner flakes of graphene nanosheets^30^,^31^,^32^. Similar findings in the Raman spectra of multilayered graphene nanosheets were also reported in literatures^29^,^30^,^31^,^32^. Hence it was confirmed that the synthesized Ag nanoparticles decorated graphene were multilayered. Raman spectra of WKF and WKF with Fe-CNTs synthesized on its surface are shown in Fig. 4(b). The peaks observed for were in accordance with the literature^33^. The synthesis of Fe-CNTs on the surface of PPy-coated WKF resulted in new peaks at 937 and 970 cm^−1^ due to quinoniod bipolaron and polaron structures of PPy, and peaks at 1061 and 1111 cm^−1^ associated with –CH stretching^34^. The intensity of the peaks at 1328 and 1577 cm^−1^ in the spectrum of neat WKF increased considerably in intensity after growth of Fe-CNTs on the surface of WKF due to the D and G bands of CNT, while a new peak appeared at 2665 cm^−1^ corresponding to the G′ band.

**Figure 5:**
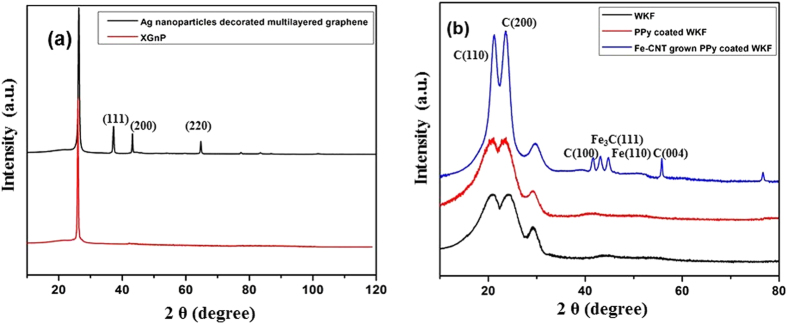
X-ray diffraction patterns of: (**a**) XGnP and Ag nanoparticle-decorated graphene, (**b**) WKF, PPy-coated WKF and Fe-CNT grown WKF.

The X-ray diffraction spectra of XGnP and Ag nanoparticle-decorated graphene are shown in Fig. 5(a). XGnP shows a sharp and single peak at 2θ = 26.35°. The Ag nanoparticle-decorated multilayered graphene shows the characteristic peak of graphene at 26.23° (002) that denoted high crystallinity of Ag-graphene as in pristine graphite nanoplatelets. The presence of the characteristic peak of graphite in the spectra of Ag-graphene might be due to some of the graphitization behaviour of graphene sheets. Generally, the thin films with less thickness have low melting temperature and the films turn out to be very unstable at high temperature^35^. During the synthesis of multilayered graphene nanosheets, the high temperature inside the microwave oven might cause assemblage of some of the graphene nanosheets into other 3D structures or segregation into islands^36^. New peaks appeared at 2θ = 37.8°, 43.5°, and 64.7° that correspond to the (111), (200) and (220) planes of cubic Ag. Yun *et al*. also synthesized Ag nanoparticles decorated graphene and reported a similar finding in the XRD analysis of the spectra^37^. Figure 5(b) shows the diffraction pattern of WKF, PPy-coated WKF, and Fe-CNT grown WKF. The characteristic peaks of WKF appeared at 2θ = 20.94° (110) and 23.98° (200)^38^. Neat PPy exhibits a broad peak between 20° and 25°. The peaks at 2θ = 20.94° (110) and 23.98° (200) in the diffraction pattern of PPy-coated WKF appear to have increased in intensity as the peaks of WKF merged with those of PPy in the diffractograms. The appearance of small new peaks at 2θ = 41.63° ((100) plane of C), 43.13° ((111) plane of Fe_3_C), 44.83° ((110) plane of Fe), 55.81° ((004) plane of CNT), and 76.61°((110) plane of CNT) in the spectrum of Fe-CNT grown PPy-coated WKF, in combination with the increased intensity of the peaks at 2θ = 20.94°and 23.98° confirmed the growth of Fe-CNTs on the surface of PPy-coated WKF^25^.

**Figure 6:**
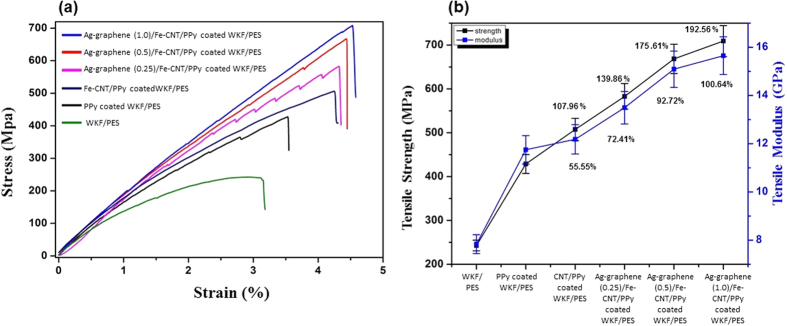
Tensile stress -strain curves (**a**) and tensile strength and modulus (**b**) of WKF/PES, PPy-coated WKF/PES, Fe-CNT grown on PPy-coated WKF/PES, Ag-graphene (0.25)/Fe-CNT/PPy-coated WKF/PES, Ag-graphene (0.5)/Fe-CNT/PPy-coated WKF/PES, Ag-graphene (1.0)/Fe-CNT/PPy-coated WKF/PES.

The stress-strain curves, tensile strengths and moduli of the WKF composites, to evaluate the degree of reinforcement provided by Fe-CNTs and Ag-graphene, are shown in Fig. 6(a,b). The tensile strength and modulus of the composites followed the order: WKF/PES < PPy-coated WKF/PES < Fe-CNT grown on PPy-coated WKF/PES < Ag-graphene (0.25)/Fe-CNT/PPy-coated WKF/PES < Ag-graphene (0.5)/Fe-CNT/PPy-coated WKF/PES < Ag-graphene (1.0)/Fe-CNT/PPy-coated WKF/PES. PPy-coated WKF exhibited a higher tensile strength than neat WKF/PES composites. The microwave-induced growth of Fe-CNTs provided additional reinforcement, resulting in high mechanical properties of the composites. Fe-CNTs were responsible for forming a networked structure in the composites, thereby increasing the interfacial interaction between fibres and polymer and thus enhancing the load transfer efficiency. The presence of Fe at the tip of the CNTs also contributed to a further increase in the interfacial area of interaction. The synthesis of Fe-CNTs resulted in a 107.96% increase in tensile strength and 55.55% increase in modulus. The inclusion of Ag nanoparticle-decorated multilayered graphene nanosheets in the composites by mixing with the PES further enhanced the mechanical properties. The high surface area of the planar wrinkled graphene nanosheets with Ag nanoparticles on their surface facilitated a greater degree of interaction between the fibre and matrix, augmenting their interlocking^39^. The Ag nanoparticles was also responsible for the enhanced mechanical interlocking within the fibers and the polymer matrix as it adhered into the resin causing local stiffening effect at the interfacial area^40^. Moreover, the improved interactions also provided efficient load transfer upon stress application. The mechanical properties further improved with an increase in the percentage of Ag-graphene, with the tensile strength increasing by 192.56% and the modulus by 100.64% for Ag-graphene (1.0)/Fe-CNT/PPy-coated WKF/PES composites.

**Figure 7:**
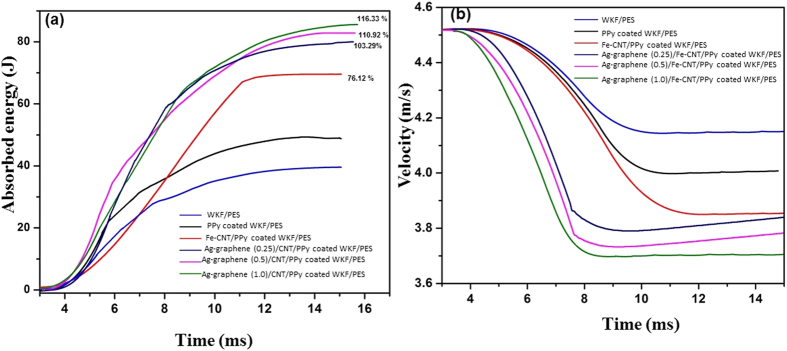
Energy-time response (**a**) and velocity-time response (**b**) curves of WKF/PES, PPy-coated WKF/PES, Fe-CNT grown on PPy-coated WKF/PES, Ag-graphene (0.25)/Fe-CNT/PPy-coated WKF/PES, Ag-graphene (0.5)/Fe-CNT/PPy-coated WKF/PES, Ag-graphene (1.0)/Fe-CNT/PPy-coated WKF/PES.

The impact resistance of the WKF composites was determined from the plot of absorbed energy vs. time as depicted in Fig. 7(a). The impact energy is the combination of the absorbed energy and rebounded energy in case of a low velocity impact. The amount of energy distributed in the composites on impact cessation is the absorbed energy. Both the bending strain energy and delamination energy constitute absorbed energy. WKF/PES composites displayed the lowest impact resistance of all composites. PPy-coated WKF/PES samples exhibited increased impact resistance compared to neat WKF/PES samples. The Fe-CNT grown PPy-coated WKF/PES samples had a 76.12% increase in impact resistance compared to neat WKF/PES. This increase was attributed to the networked structure after growth of Fe-CNTs and the higher interfacial interaction provided by both the Fe nanoparticles and the CNTs between fibres and matrix. Taraghi *et al*. previously reported an improved impact resistance after the incorporation of MWCNTs into woven Kevlar/epoxy composites^41^. Additional surface area provided by Ag nanoparticle-decorated graphene nanosheets facilitated further interactions and allowed even higher energy absorption by the Ag-graphene Fe-CNT/PPy-coated WKF/PES samples. The highest energy absorption (a 116.33% increase over neat WKF/PES) was observed with Ag-graphene Fe-CNT/PPy-coated WKF/PES samples having a 1.0% Ag-graphene content. The higher energy absorption was attributed to combined properties of both Ag nanoparticles and multilayered graphene nanosheets. The incorporation of Ag nanoparticles prevented aggregation of multilayered graphene sheets by acting as a separator. The Ag nanoparticles adhered to the surface of the graphene nanosheets amplified the adhesion contact points and thus enhanced network association of graphene, polymer matrix and fiber. Ag-graphene restricted the distortion sliding efficiently and accumulated the dislocations at the interfacial region under the influence of impact. Li *et al*. reported improvement in mechanical properties after incorporation of Ni nanoparticles decorated graphene nanoplatelets in Cu matrix composite^42^.

The velocity-time response curves of the composites are presented in Fig. 7(b). The difference between incident and residual velocity, defined as the penetration limit, was determined for all composites. The values were the highest for samples incorporating Ag nanoparticle-decorated graphene. The penetration limit was the lowest for WKF/PES composites, with PPy-deposited WKF showing a higher penetration limit than neat WKF/PES. Fe-CNTs increased stiffness of the composites, leading to higher penetration limit values for Fe-CNT/PPy-coated WKF/PES samples. Incorporation of Ag-graphene along with Fe-CNTs induced significant additional stiffness due to the high surface area and superior interfacial interaction. The combined effect of Fe-CNTs and Ag-graphene in the composites promoted a higher rate of decrease in velocity, thus making them exceptionally tough.

**Figure 8:**
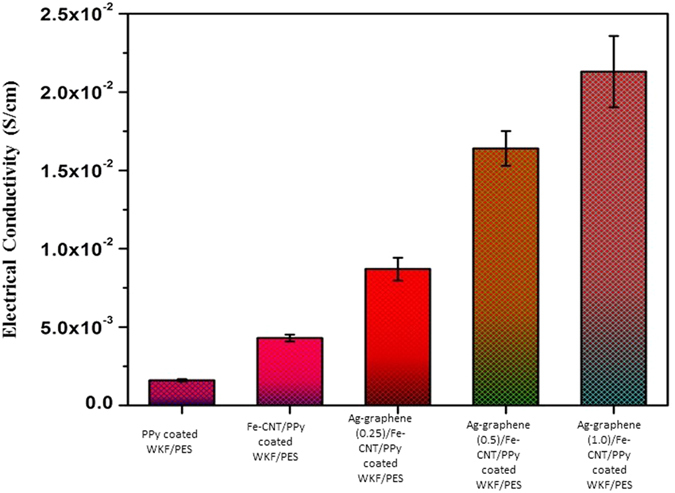
Electrical conductivity of PPy-coated WKF/PES, Fe-CNT grown on PPy-coated WKF/PES, Ag-graphene (0.25)/Fe-CNT/PPy-coated WKF/PES, Ag-graphene (0.5)/Fe-CNT/PPy-coated WKF/PES, Ag-graphene (1.0)/Fe-CNT/PPy-coated WKF/PES.

The electrical conductivity values of the WKF composites are shown in Fig. 8. PPy-coated WKF/PES composites showed electrical conducting behaviour comparable to the insulating WKF/PES composites. The synthesis of Fe-CNTs on the surface of PPy-coated WKF/PES enhanced the electrical conductivity of the composites, caused by both Fe nanoparticles and CNT. Liu *et al*. prepared PPy-coated fly ash, exposed it to microwave radiation to synthesize CNT, and reported that CNT improved the electrical conductivity^43^. However, in our study, Fe-CNT grown PPy-coated WKF/PES composites displayed a relatively low increase in electrical conductivity due to the presence of PES resin in the composites. Similar findings were also reported for polyaniline-coated glass fibre/isotactic polypropylene/maleic anhydride grafted polypropylene composites^44^. The synergistic effect of Ag nanoparticle-decorated multilayered graphene nanosheets and Fe-CNTs in Ag-graphene/Fe-CNT/PPy-coated WKF/PES composites further improved their electrical conductivity. This enhancement was ascribed to the presence of Ag, the most conductive metal. The Ag nanoparticles improved the electrical conductivity by increasing the electron transport ability between the layers of graphene nanosheets^45^. The conductivity of the composites increased with an increase in the percentage of Ag-graphene.

**Figure 9:**
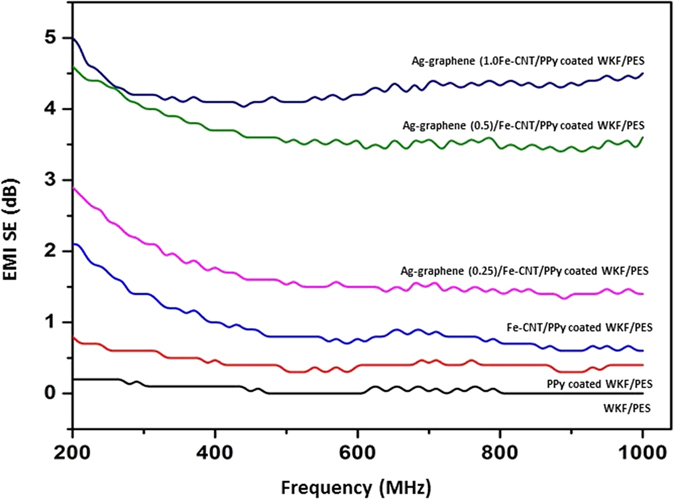
Total electromagnetic interference (EMI) shielding effectiveness (SE) of WKF/PES, PPy-coated WKF/PES, Fe-CNT grown on PPy-coated WKF/PES, Ag-graphene (0.25)/Fe-CNT/PPy-coated WKF/PES, Ag-graphene (0.5)/Fe-CNT/PPy-coated WKF/PES, and Ag-graphene (1.0)/Fe-CNT/PPy-coated WKF/PES.

The total EMI shielding effectiveness (SE) of each WKF composite is revealed in Fig. 9. The total EMI SE is a combination of the reflection (SE_R_), absorption (SE_A_) and multiple-reflection (SE_M_) SEs of EM radiation incident on the surface of a material. The impedance between the shielding material and air is related to the reflection, the energy dissipation of absorbed incident EM waves resulted in absorption, and the non-uniformity of the interior of the shielding composite panel resulted in multiple reflection. The multiple reflection is taken onto account only in case of porous or foam material where there is large surface areas^46^. Thus, the EMI SE of a material can be expressed as^47^,^48^:


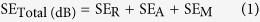



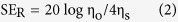



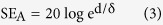



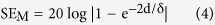



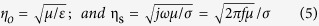


where *μ, σ* and *ε* denote the magnetic permeability, electrical conductivity and permittivity of the medium, respectively, and *ω* and *f* represent the angular frequency of the radiation and frequency of the electromagnetic wave, respectively. From the above equations, it is clear that electrical conductivity is one of the main parameters for reflection suppression and a major component in shielding by absorption^48^. Hence, decorating highly conducting Ag nanoparticles on graphene sheets and Fe nanoparticles on the tips of CNTs might enhance the absorption of incident EM waves. The sample thickness is a significant contributor to a high EMI SE, since the absorption, reflection as well as multiple reflection SEs all increase with an increasing thickness. For example, the absorption attenuation increases due to the increased presence of efficient materials. Therefore, massive, thick materials have enhanced absorption attenuation in EMI shielding^49^.

Here, the thickness of all the samples was 0.35 mm. Since the fabricated composites were very thin and prepared with only a single layer of plain weave WKF, low SE values were obtained for all of them. The SE value at 0.20 dB was the lowest for neat WKF/PES composites. Deposition of conducting PPy polymer on WKF resulted in a higher total EMI SE for PPy-coated WKF/PES composites than for neat WKF/PES composites. The Fe-CNTs synthesized on the surface of PPy-coated WKF had a significant effect, enhancing the EMI SE of Fe-CNT/PPy-coated WKF/PES composites. This was mainly ascribed to the arrangement of the conducting interconnected networks by Fe-CNTs in the PPy-coated WKF/PES composites. The interrelated networks of CNTs and ferromagnetic Fe nanoparticles in the composite interacted with the incident EM radiation, thereby acting as a shield and leading to the enhanced SE^50^. The SE of metal nanoparticle-decorated CNTs was previously shown to increase due to the introduction of magnetic permeability that increases EM wave absorption^51^. Co/Ni nanoparticles attached to single-walled CNTs exhibited similar effective microwave shielding behavior^52^. Here, Ag nanoparticle-decorated multilayered graphene contributed to an additional SE, and the overall SE increased with an increase in Ag-graphene content. The incident EM waves entering the composites were dissipated by conductive Ag nano particles that resulted in effective EMI shielding of the composites^53^. Moreover, the presence of abundant active atoms on the surface of the Ag nanoparticles due to their low density and higher specific surface area promoted interfacial polarization that resulted in a large interface dielectric loss. Thus, Ag nanoparticles exhibited large electric loss and showed promising EMI shielding^54^. The decoration of Ag nanoparticles on the surface of graphene nanosheets merged the properties of both the constituents effective for EMI shielding. The multilayered graphene nanosheets interact with incoming EM waves and shield the composites from them. Shielding by absorption increases when electrical or magnetic dipoles present in the shielding composite material interact with the EM radiation. It has been found that increasing the Ag-graphene amount enhanced the number of connections between the Ag nanoparticles decorated graphene sheets and hence rising the conductivity networks in the composite. Samples treated with 1.0% Ag-graphene showed the highest EMI SE of 5 dB (a 25-fold increase compared to neat WKF/PES composites). Ag nanoparticles may also offer reflective properties due to their high electrical conductivity^55^. Previously, a total EMI SE of 3.3 dB was measured when preparing MWCNT/polypropylene (0.34 mm thickness) composite plates with 2.5 vol.% MWCNTs^47^. Although an improvement in EMI SE was achieved for the WKF/PES composites here, further detailed studies are required to investigate the effect of different nanostructures for composites with higher thicknesses.

## Conclusion

Fe nanoparticle-decorated carbon nanotubes (Fe-CNT) were grown on polypyrrole (PPy) deposited on woven Kevlar fibre (WKF), and composites of the Fe-CNT grown WKF and Ag-graphene dispersed in polyester resin (PES) were then fabricated by a vacuum-assisted resin transfer moulding technique. A microwave-induced technique was utilized for the synthesis of both Fe-CNTs and Ag nanoparticle-decorated multilayered graphene nanosheets, which is a very economical and time saving approach. WKF was surface functionalized and PPy was *in-situ* polymerized on the surface of WKF. The direct synthesis of Fe-CNTs on the fibres prevented the problem of agglomeration. Scanning electron microscopy showed the growth of CNTs with Fe nanoparticles at their tips on the surface of WKF, as well as the decoration of multilayered graphene nanosheets with Ag nanoparticles. The Fe-CNTs and Ag-graphene were further characterized by Raman spectroscopy and X-ray diffraction analysis. The tensile strength and modulus of PPy-coated WKF/PES composites was enhanced by the growth of Fe-CNTs on their surface, while an increase in the amount of Ag-graphene further enhanced the mechanical properties of the composites. The highest increase in tensile strength (192.56%) and modulus (100.64%) was achieved with 1% Ag-graphene in the composites. A significant improvement in absorbed impact energy and penetration limit of the Ag-graphene/Fe-CNT/PPy-coated WKF/PES compared to neat WKF/PES was observed due to the synergistic reinforcing effects of Fe-CNTs and Ag-graphene. Fe-CNT/PPy-coated WKF/PES composites with 1% Ag-graphene displayed the highest impact resistance and an absorbed energy increase of 116.33%. The *in-situ* polymerization of pyrrole induced some conductivity in WKF/PES, but the combined effect of Fe-CNTs and Ag-graphene increased it to a remarkable extent. Finally, the total electromagnetic interference (EMI) shielding effectiveness (SE) value of a single sheet of neat WKF/PES was 0.20 dB, butwas25-fold higher for Fe-CNT/PPy-coated WKF/PES composites containing 1% Ag-graphene. The higher the Ag-graphene content, the higher the total EMI SE of the composites. Although an increase in EMI shielding properties was observed, more studies are required to investigate the nanofiller effects for thick composites prepared with multilayered sheets of WKF. Nonetheless, the improved interaction and formation of a conductive network between the WKF, PPy, Fe-CNT, Ag-graphene and PES in the composites contributed to their enhanced mechanical, electrical and EMI shielding properties in this study.

## Methods

Woven Kevlar fibres (WKF) (Kevlar 49) were obtained from JMC Co., Ltd. (Gyeongsangbuk-do, Korea). Microwave Research & Applications Inc. USA (BP110 laboratory grade microwave oven) supplied a laboratory scale microwave oven. Silver nitrate was obtained from Daejung Chemicals and Metals Co. Ltd. Korea and graphite nanoplatelets from Hanwha Nanotech Corp, Korea. Pyrrole, ferric chloride hexahydrate (FeCl_3_.6H_2_O), ferrocene, hexane and toluene were purchased from Sigma-Aldrich (St. Louis, MO, USA). Duksan pure chemicals, Korea, supplied reagent grade hydrogen peroxide. PES (LSP-8020B) and methyl ethyl ketone peroxide were obtained from CCP Composites and ARKEMA, Korea. Acetone and ethanol was supplied by J.T. Baker (Phillipsburg, NJ, USA) and used as received.

### *In-situ* oxidative polymerization of pyrrole on WKF

Methanol and water were mixed in a 50:50 ratio and 12% v/v pyrrole was added. WKF samples were soaked in the pyrrole solution for 60 min at room temperature and then placed in a sonicator bath. The samples were taken out after 60 min and put in an aqueous solution of FeCl_3_.6H_2_O (12% w/v), which was used as the oxidizing agent. WKF samples were taken out of the FeCl_3_.6H_2_O solution after 15 min for complete polymerization of pyrrole. Finally, PPy-coated WKF samples were washed several times with deionized water and dried overnight in an oven at 60 °C.

### Microwave-assisted synthesis of Fe-CNT

A ferrocene solution was prepared by dissolving ferrocene in toluene, and the PPy-coated WKF samples were placed in this solution at a 1:1 ratio of sample to ferrocene. The samples were then placed in a sonicator bath for 15 min for good distribution of ferrocene throughout the samples. The growth of Fe-CNTs on the surface of the fibres was enhanced by the addition of 0.25 mL of hexane. After leaving the samples in air for 1 min to let the solvents evaporate, they were exposed to microwave irradiation.

### Microwave-assisted synthesis of Ag-graphene

Natural graphite, hydrogen peroxide and ammonium persulfate were mixed in a glass bottle in a 2:1:0.1 weight ratio, and exposed to microwave irradiation for 30 s. Then, urea and ammonia were added as reducing agents, and 20 mL of 1 N silver nitrate was added to the mixture as the metal precursor. The mixture was then sonicated for 1 h and subjected to microwave irradiation for 90–100 s. The precursors exfoliated rapidly, followed by some arc and lightning on exposure to microwave irradiation. The resulting Ag-graphene was dispersed in unsaturated PES by sonication before the preparation of the composites through VARTM^23^.

### Characterization

A Nova Nano SEM 230 (FEI, Hillsboro, OR, USA) was operated at 15 kV to investigate the growth of Fe-CNTs on the surface of WKF and Ag-graphene. Normal TEM (JEM-2100, JEOL) was used to characterize the growth of Fe-CNT and Ag-graphene. A micro-Raman Microscope (WITec) was used using 532 nm excitation via the back scattering geometry with the polarizer and analyzer parallel to one another to obtain the Raman spectra. The X-ray diffraction patterns of the samples were studied using a wide-angle X-ray diffractometer (Bruker, Billerica, MA, USA) at a 40 kV operating voltage with a 20 mA current using crystal-monochromated Cu–Kα radiation in the range of 5°–80° (2θ). Tensile tests for the samples were performed with an Instron 5982 universal testing machine at a displacement rate of 2 mm/min with a maximum load of 100 kN following ASTM D3039 standard. The results for each composite were an average of three specimens. The impact test was carried out using a drop-weight impact tester (model 5982; Instron, Norwood, MA, USA) in accordance with the ASTM D5628-10 standard. The circular clamp was loaded with a 5-kg impactor with a 40 mm diameter, and data were collected between the initial impact contact and penetration points. 200 J of energy was applied to all the samples to fully perforate them. Electrical conductivity tests were performed with a 6517 multimeter (Keithley Instruments, Beachwood, OH, USA). The EMI SEs of the composites were measured using an EMI SE test system (Rhode and Schwartz; Munich, Germany). The tests were carried out in accordance with ASTMD4935, utilizing a coaxial cable with type-N connectors. The frequency was measured up to 1 GHz, and the attenuation of EM radiation was obtained as a function of frequency.

## Additional Information

**How to cite this article**: Hazarika, A. *et al*. Microwave-synthesized freestanding iron-carbon nanotubes on polyester composites of woven Kevlar fibre and silver nanoparticle-decorated graphene. *Sci. Rep.* **7**, 40386; doi: 10.1038/srep40386 (2017).

**Publisher's note:** Springer Nature remains neutral with regard to jurisdictional claims in published maps and institutional affiliations.

## Figures and Tables

**Figure 1:**
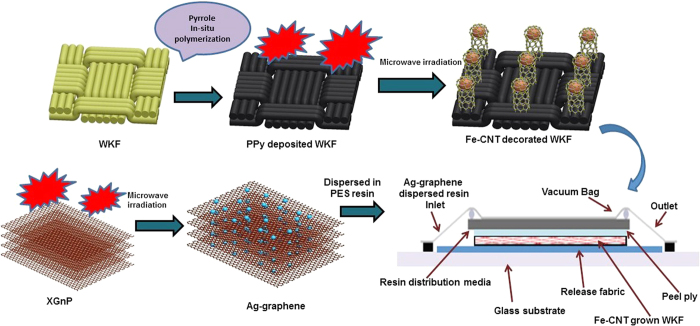
Schematic diagram of the preparation of Ag-graphene/Fe-CNT/PPy-coated WKF/PES composites. Fe nanoparticle-decorated carbon nanotubes (Fe-CNT) were grown on polypyrrole (PPy) deposited on woven Kevlar fibre (WKF) using microwave irradiation. Composites of the Fe-CNT grown WKF and Ag-graphene dispersed in polyester resin (PES) were then fabricated by vacuum-assisted resin transfer moulding.
